# RETRACTION: *Withania Frutescens*. *L* Extract: Phytochemical Characterization and Acute and Repeated Dose 28‐Day Oral Toxicity Studies in Mice

**DOI:** 10.1155/bmri/9842361

**Published:** 2026-01-09

**Authors:** BioMed Research International

RETRACTION: A.EL. Moussaoui, M. Bourhia, F.Z. Jawhari, I. Es‐safi, S.S. Ali, A. Bari, H.M. Mahmood, D. Bousta, A. Bari, “*Withania frutescens. L* Extract: Phytochemical Characterization and Acute and Repeated Dose 28‐Day Oral Toxicity Studies in Mice,” *BioMed Research International*, 2020, https://doi.org/10.1155/2020/1976298

The above article, published online on 27 September 2020 in Wiley Online Library (http://wileyonlinelibrary.com), has been retracted by agreement between the journal Section Editor, Jinsong Ren; and John Wiley & Sons Ltd. The retraction has been agreed due to concerns with Figures 6, 7 and 8. Specifically:

• Unexpected areas of similarity between the Control and 2000 mg/kg panels of Figure 7
• Unexpected areas of similarity between the Control and 500 mg/kg panels of Figure 6
• Unexpected areas of similarity between the 500 mg/kg and 2000 mg/kg panels of Figure 6
• Unexpected areas of similarity between the Control and 500 mg/kg panels of Figure 8
• Unexpected areas of similarity between the Control and 2000 mg/kg panels of Figure 8
• The Control panel of Figure 6 is identical to the Control panel of Figure 6 in [1]
• Unexpected areas of similarity between the 2000 mg/kg panel of Figure 7 and the Control panel of Figure 8 in [1]
• Unexpected areas of similarity between the 500 mg/kg panel of Figure 6 and the Control panel of Figure 6 in [1]
• The Control panel of Figure 7 is identical to the Control panel of Figure 8 in [1]
• The Control panel of Figure 8 is identical to the Control panel of Figure 7 in [1]
• Unexpected areas of similarity between the 500 mg/kg panel of Figure 8 and the Control panel of Figure 7 of [1]

Additionally, the below figure concerns were identified with publications that were published after this article:

• The Control panel of Figure 7 shows high similarity to Figure 2e (stretched) in [2]
• The Control panel of Figure 8 is identical to Figure 2c in [2]
• The Control panel of Figure 7 shows high similarity to the Kidney/Control panel in Figure 4 of [3]

After assessment of the author’s response and the figure concerns, the editorial board no longer finds the results presented in the article reliable, and it is retracted.

M. Bourhia agrees to the retraction, whilst no response was received from the other authors.
